# Visualization of wrist anatomy—a comparison between 7T and 3T MRI

**DOI:** 10.1007/s00330-021-08165-5

**Published:** 2021-08-11

**Authors:** Simon Götestrand, Anders Björkman, Isabella M. Björkman-Burtscher, Ingvar Kristiansson, Elenya Aksyuk, Pawel Szaro, Karin Markenroth Bloch, Mats Geijer

**Affiliations:** 1 Department of Medical Imaging and Physiology, Skåne University Hospital, Lund, Sweden; 2 Department of Clinical Sciences, Faculty of Medicine, Lund University, Lund, Sweden; 3 Department of Hand Surgery, Institute of Clinical Sciences, Sahlgrenska Academy, University of Gothenburg, Gothenburg, Sweden; 4 Sahlgrenska University Hospital, Region Västra Götaland, Gothenburg, Sweden; 5 Department of Radiology, Institute of Clinical Sciences, Sahlgrenska Academy, University of Gothenburg, Gothenburg, Sweden; 6 Department of Radiology, Sahlgrenska University Hospital, Region Västra Götaland, Gothenburg, Sweden; 7 Lund University Bioimaging Center, Lund University, Lund, Sweden

**Keywords:** Carpal joints, Triangular fibrocartilage, Ligaments, Cartilage, articular, Area under curve

## Abstract

**Objective:**

Injuries to the wrist are, due to its small size and complex anatomical structures, difficult to assess by MR, and surgical interventions such as diagnostic arthroscopy are often necessary. Therefore, improved visualization using non-invasive methods could be of clinical value. As a first step of improvement, the purpose of this study was to evaluate visualization of anatomical structures at 7T compared with 3T MR.

**Methods:**

Eighteen healthy volunteers (three males and three females from each age decade between 20 and 49 years) were examined with 7T and 3T MR. Four musculoskeletal radiologists graded 2D and 3D images on a five-level grading scale for visibility of ligaments, cartilage, nerves, trabecular bone, and tendons, as well as overall image quality (i.e., edge sharpness, perceived tissue contrast, and presence of artefacts). Statistical analysis was done using a visual grading characteristics (VGC) analysis.

**Results:**

Visibility of cartilage, trabecular bone, tendons, nerves, and ligaments was graded significantly higher at 7T with an area under the curve (AUC_VGC_) of 0.62–0.88 (95% confidence interval [CI] 0.50–0.97, *p* = < 0.0001–0.03) using either 2D or 3D imaging. Imaging with 3T was not graded as superior to 7T for any structure. Image quality was also significantly superior at 7T, except for artefacts, where no significant differences were found.

**Conclusions:**

Tendons, trabecular bone, nerves, and ligaments were all significantly better visualized at 7T compared to 3T.

**Key Points:**

• *MRI of the wrist at 7T with a commercially available wrist coil is feasible at similar acquisition times as for 3T MRI.*

• *The current study showed 7T to be superior to 3T in the visualization of anatomical structures of the wrist, including ligaments, tendons, nerves, and trabecular bone.*

• *Image quality was significantly superior at 7T, except for artefacts, where no significant differences were found.*

**Supplementary Information:**

The online version contains supplementary material available at 10.1007/s00330-021-08165-5.

## Introduction

The anatomy of the wrist, including the radiocarpal joint, the distal radioulnar joint, the intracarpal joints, and interconnecting ligaments, is complex. In addition, a large number of flexor and extensor tendons and three major nerves are located around the wrist. All wrist structures are prone to injury. The small and intricate anatomy of these structures makes them difficult to assess using MR [1]. Thus, wrist arthroscopy is still considered the reference standard for assessing intra-articular cartilage and ligament injuries in the radiocarpal and intercarpal joints [1–3]. Improvement of a non-invasive method, such as MRI, in the evaluation of these structures could be valuable, as wrist arthroscopy is not only invasive but also expensive and comes with the risk of complications [4]. Additionally, some structures such as nerves and tendons are not possible to assess using an arthroscopic technique. Improved visualization of ligaments, nerves, tendons, and trabecular bone would, therefore, be of clinical value [5–8].

Previous studies have reported 3T systems to be superior to 1.5T at depicting ligament structures of the wrist [3–9], especially when using the improved signal-to-noise ratio (SNR) to increase spatial resolution [10]. 3T imaging is now widely used in clinical musculoskeletal practice. As ultra-high field (UHF) MR systems, 4T and above, have become increasingly available for research and clinical purposes, their potential for improving clinical diagnostics has increased. A higher field strength provides a higher intrinsic SNR which brings potentially higher resolution and potentially improved tissue contrast compared to lower field strengths [11]. Initial experiences in anatomical imaging of the wrist at 7T are promising [10, 12], but the number of published studies in this field is very limited [5–7, 10, 12–18]. In addition, the lack of commercially available, dedicated radio frequency (RF) wrist coils for 7T has earlier been a technical challenge for imaging at 7T [10]. No study has been published on the use of a commercially available wrist coil specifically developed for wrist imaging at 7T. The benefit in SNR at 7T compared to 3T has been established, but improved SNR does not necessarily improve the visibility of anatomic structures [15].

The purpose of this study was to investigate whether MR at 7T, using a dedicated, commercially available wrist coil, improves anatomical visualization of clinically important structures in the wrist such as ligaments, cartilage, nerves, tendons, and trabecular bone compared to imaging at 3T.

## Material and methods

### Study design

The appropriate ethics committee approved this single-center prospective study (2017/193) which was carried out in accordance with the Declaration of Helsinki. All subjects gave written informed consent.

### Subjects

The study included 18 healthy subjects, with three male and three female subjects from each decade between 20 and 49 years. The inclusion criterion was a normal clinical hand status, confirmed by a clinical hand and wrist examination performed by a hand surgeon with over 20 years of clinical experience. The clinical examination included a stability test of the distal radioulnar joint; palpation of the soft spot and examination of the extensor carpi ulnaris (ECU) tendon over the proximal part of the carpus and the distal part of the ulna to evaluate the triangular fibrocartilage complex (TFCC) [19]; Watson’s shift test [20] to assess the scapholunate ligament (SLL); the lunotriquetral (LT) shuck test, as described by Kleinman [19], to evaluate the LT ligament (LTL); assessment of median and ulnar nerve sensory and motor function including Tinel’s test over the carpal tunnel and Guyon’s canal besides Phalen’s test [21]; individual assessment for function and synovitis of all flexor and extensor tendons; and assessment of grip strength [21].

Exclusion criteria were contraindications for MR, subjective symptoms from the right wrist, history of injury to the right wrist or hand, or inability to understand written or spoken instructions in Swedish.

### MR imaging

MR was performed using an actively shielded 7T MR scanner (Achieva, Philips) with a single-channel transmit and 16-channel receive wrist coil (RAPID Biomedical) and a 3T MR scanner (Magnetom Skyra, Siemens Healthineers) with a 16-channel receive wrist coil (Siemens Healthineers). The right wrist and adjoining parts of the forearm and hand were examined. The intention was to keep the time span between the two examinations as short as possible, and the median interval between the 7T and 3T examinations was 3 days. For 7T MR, the protocol comprised a three-dimensional (3D) proton density (PD) turbo spin echo (TSE) sequence, with a spatial resolution of 0.45 × 0.45 × 0.45 mm^3^; two-dimensional (2D) fat-saturated (FS) PD-weighted sequences acquired in three orthogonal planes, with a spatial resolution of 0.35 × 0.36 × 1.2 mm^3^; and a coronal T1-weighted sequence, with a spatial resolution of 0.3 × 0.3 × 2.0 mm^3^ (Supplementary Table 1). The total acquisition time for all sequences was 20:18 min. For 3T MR, the protocol comprised a 3D PD-weighted “sampling perfection with application-optimized contrasts using different flip angle evolution” (SPACE) sequence acquired in the coronal plane, with a spatial resolution of 0.5 × 0.5 × 0.5 mm^3^, a 2D FS PD-weighted sequence acquired in three orthogonal planes, with a spatial resolution of 0.3 × 0.3 × 2.0 mm^3^ in the coronal and axial plane, and 0.3 × 0.3 × 3.0 mm^3^ in the sagittal plane and a coronal T1-weighted sequence, with a spatial resolution of 0.3 × 0.3 × 2.0 mm^3^ (Supplementary Table 2). The total acquisition time for all sequences was 23:33 min. A line between the volar border of the scaphoid and the pisiform, drawn in the axial plane, defined the coronal plane. The 3D PD sequences were optimized for visualization of ligaments for both 7T and 3T.

### Image evaluation

All image data were pseudonymized. 3T and 7T examinations were randomized and four observers, with 28, 18, 5, and 1.5 years’ experience as musculoskeletal radiologists, independently graded each examination separately. Thus, the study comprised 36 data sets that were presented in a randomized order for each observer. A grading scale with five levels was used for image evaluation of anatomical visibility and image quality (Table 1). The anatomical structures evaluated are listed in Table 2 together with a description of the anatomical level they were graded at and the sequence(s) used. Image grading was performed on a clinical IDS7 PACS station (SECTRA AB). When evaluating ligaments using 2D sequences, the grading represented the plane with the best visualization of the structure. When evaluating structures using a 3D sequence, each observer created their own multiplanar reformations (MPR) in the PACS MPR application optimized for visualization of the structure. Image quality parameters were edge sharpness, perceived tissue contrast, and presence of artefacts.

**Table. 1:** Definition of outcome parameters for image evaluation

Scored parameters	
Anatomical structure	Image quality
SLL dorsal and palmar portion	Edge sharpness
LTL dorsal and palmar portion	Perceived tissue contrast
TFCC radial, ulnar and foveal attachment	Artefacts
Articular cartilage between the triquetral and hamate bone	
Median nerve	
Ulnar nerve	
ECU tendon	
Bone trabeculae of the capitate bone	
Scoring definitions	
Not visible (Score 1)	Unacceptable (Score1)
Visible, but with complete loss of detail (Score 2)	Inadequate (Score 2)
Visible, with visualization of some anatomical detail (Score 3)	Adequate (Score 3)
Visible, with visualization of most anatomical detail (Score 4)	Good (Score 4)
Visible, with perfect visualization of anatomical detail (Score 5)	Excellent (Score 5)

*ECU*, extensor carpi ulnaris; *LTL*, lunotriquetral; *SLL*, scapholunate; *TFCC*, triangular fibrocartilage complex

**Table. 2:** Evaluated anatomical structures, detailing at which anatomical level they were graded, and which MR sequence(s) was/were used. Each observer freely chose the MPR planes used

Anatomical structure	Anatomical level	MR sequences		
		2D PD FS	3D PD FS	T1
TFCC	The radial, ulnar styloid and foveal attachments	Axial Sagittal Coronal	MPR	
SLL	The dorsal and palmar portion	Axial Sagittal Coronal	MPR	
LTL	The dorsal and palmar portion	Axial Sagittal Coronal	MPR	
Articular cartilage	Between the triquetral and hamate bone	Coronal	Coronal MPR	
Median nerve	At the level of the pisiform bone	Axial	Axial MPR	
Ulnar nerve	At the level of the pisiform bone	Axial	Axial MPR	
ECU tendon	At the dorsal groove of the ulnar head	Axial	Axial MPR	
Bone trabeculae	Of the capitate bone			Coronal

*2D*, two-dimensional; 3D, three-dimensional; *ECU*, extensor carpi ulnaris; *FS*, fat saturated; *LTL*, lunotriquetral ligament; *MPR*, multiplanar reformations; *PD*, proton density; *SLL*, scapholunate ligament; *TFCC*, triangular fibrocartilage complex

Before grading, one of the authors held a training session with all observers to ensure conformity in evaluation, using images from a previous study carried out by four of the authors, and images produced during sequence optimization. Images representing all five grades on the scale for all structures were presented. Subsequently, all sequences from three test subjects, not included in the study, were examined and graded collectively. The observers were provided with example images of all structures for each grade (Table 1) to use as a reference during grading, and the observers were free to use their preferred window and level settings, magnification, and scrolling mode.

### Statistics

Categorical variables are presented as frequencies and percentages. To determine whether image evaluation grades were significantly different between imaging at 7T and 3T, the Visual Grading Characteristics (VGC) Analyzer software (in-house developed software, University of Gothenburg) was used [22]. VGC analysis is a non-parametric rank-invariant method for the analysis of visual grading data [22] and the analysis was based on the trapezoid VGC curve, using fixed-reader analysis. To measure the separation between the two grading distributions for 3T and 7T MR for each graded item, the area under the VGC curve (AUC_VGC_) (0 ≤ AUC_VGC_ ≤ 1) was used. An AUC_VGC_ of 0.5 suggests that image quality on average was graded equally for the 7T and the 3T protocol. An AUC_VGC_ of > 0.5 suggests that image quality at 7T was superior. An AUC_VGC_ of < 0.5 suggests that image quality at 3T was superior. A larger intra-observer or inter-observer variability leads to a widening of the estimated confidence interval by the software’s use of a resampling technique. Therefore, intra- and inter-observer agreements affect the estimated uncertainty of the obtained figure-of-merit (the AUC_VGC_). *P*-values < 0.05 were considered statistically significant.

## Results

**Fig. 1:**
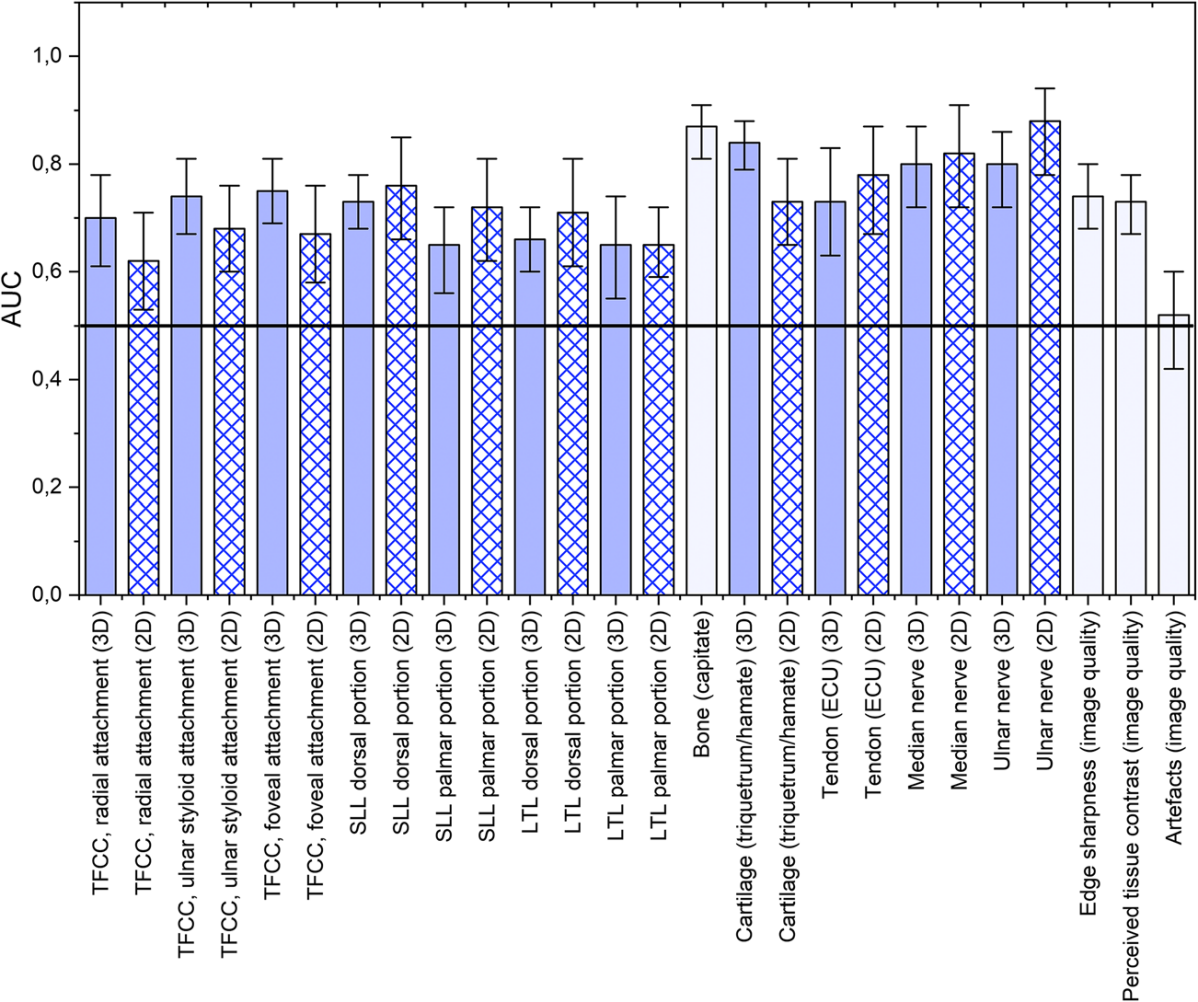
VGC analysis comparing grading between 7T and 3T imaging. If the bar showing the area under curve, AUC_VGC_ including the range bar (i.e., the 95% confidence interval) is above 0.5, 7T imaging was on average graded significantly better than 3T imaging. Wider confidence intervals imply lower observer agreement. 2D, two-dimensional; 3D, three-dimensional; AUC, area under the curve; ECU, extensor carpi ulnaris; LTL, lunotriquetral ligament; SLL, scapholunate ligament; TFCC, triangular fibrocartilage complex; VGC, visual grading characteristics. 2D, two-dimensional; 3D, three-dimensional; AUC, area under curve; ECU, extensor carpi ulnaris; LTL, lunotriquetral ligament; SLL, scapholunate ligament; TFCC, triangular fibrocartilage complex

**Fig. 2:**
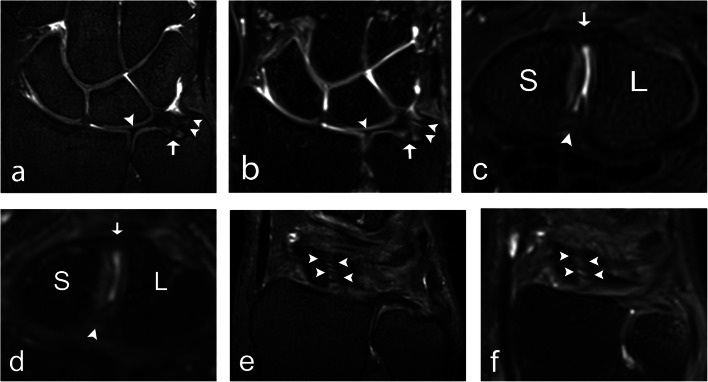
A 32-year-old male healthy volunteer (**a**, **b**), a 22-year-old male healthy volunteer (**c**, **d**), and a 27-year-old female healthy volunteer (**e**, **f**). **a** A 7 T 0.5-mm-thick coronal 3D PD TSE section and (**b**) a 3 T 0.5-mm-thick coronal 3D PD SPACE section with a depiction of the ulnar styloid attachment (arrowheads), the foveal attachment (arrow), and the radial attachment (arrowhead) of the TFCC. **c** A 7 T 0.5-mm-thick axial 3D PD TSE section and (**d**) a 3 T 0.5-mm-thick axial 3D PD SPACE section with visualization of the dorsal portion (arrow), and the palmar portion (arrowhead) of the SLL. **e** A 7 T 0.5-mm-thick coronal 3D PD TSE section and (**f**) a 3 T 0.5-mm-thick coronal 3D PD SPACE section depicts the dorsal portion of the SLL (arrowheads). 3D, three-dimensional; PD, proton density; S, scaphoid; SLL, scapholunate ligament; SPACE, “sampling perfection with application-optimized contrasts using different flip angle evolution”; TFCC, triangular fibrocartilage complex; TSE, turbo spin echo

**Fig. 3:**
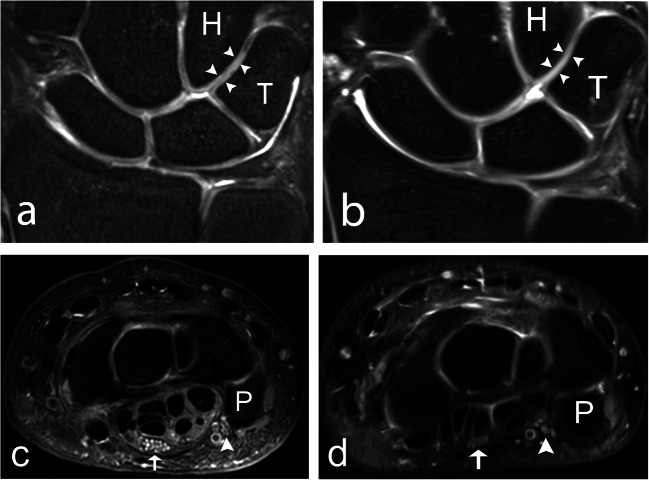
A 39-year-old male healthy volunteer. **a** A 7 T and (**b**) a 3 T coronal 2D PD-weighted section, showing the articular cartilage (arrowheads) between the hamate and the triquetrum. Articulate cartilage between other bones is also visible. **c** a 7 T and (**d**) a 3 T axial 2D PD-weighted section, showing the median (arrow), and ulnar nerve (arrowhead), at the level of the pisiform bone. 2D, two-dimensional; H, hamate; P, pisiform; PD, proton density; T, triquetrum

**Fig. 4:**
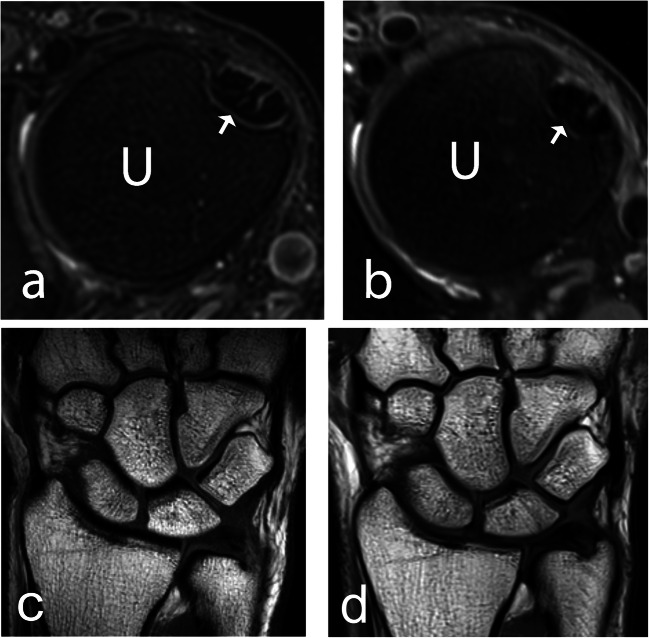
A 42-year-old male healthy volunteer (**a**, **b**) and a 29-year-old male healthy volunteer (**c**, **d**). **a** A 7 T and (**b**) a 3 T axial 2D PD-weighted section, with a depiction of the ECU tendon (arrow) at the dorsal groove of the ulnar head. **c** A 7 T and (**d**) a 3 T coronal T1-weighted section, illustrating visualization of bone structure. 2D, two-dimensional; ECU, extensor carpi ulnaris; PD, proton density; U, ulna

All evaluated anatomical structures, including ligaments, trabecular bone, cartilage, nerves, and tendons, were graded as better visualized at 7T compared to 3T (Table 3, Fig. 1). In evaluating image quality, 7T was significantly superior to 3T in the evaluation of edge sharpness and perceived tissue contrast. There was no significant difference in grading regarding artefacts. Examples of the superiority of 7T over 3T regarding anatomical visibility are given for the TFCC and the SLL in Fig. 2, for articular cartilage and the median and ulnar nerves in Fig. 3, and for the ECU tendon and trabecular bone in Fig. 4.

**Table. 3:** Visual grading characteristic analysis, comparing ratings between 7T and 3T MR. An AUC_VGC_ of 0.5 shows that the image quality on average is graded equally at 7T and 3T. An AUC_VGC_ of > 0.5 shows that the image quality is superior at 7T. An AUC_VGC_ of < 0.5 shows that the image quality is superior at 3T. If there is a statistically significant difference between grading, the confidence interval does not contain 0.5. The table also includes the median and average grades for each parameter

VGC Analysis	AUC_VGC_ (95 % CI)	*p* value
TFCC, radial attachment (3D) TFCC, radial attachment (2D)	0.70 (0.61–0.78) 0.62 (0.53–0.71)	< 0.0000001 0.011
TFCC, ulnar styloid attachment (3D) TFCC, ulnar styloid attachment (2D)	0.74 (0.67–0.81) 0.68 (0.60–0.76)	< 0.0000001 < 0.0000001
TFCC, foveal attachment (3D) TFCC, foveal attachment (2D)	0.75 (0.69–0.81) 0.67 (0.58–0.76)	< 0.0000001 0.0025
SLL dorsal portion (3D) SLL dorsal portion (2D)	0.73 (0.68–0.78) 0.76 (0.66–0.85)	< 0.0000001 < 0.0000001
SLL palmar portion (3D) SLL palmar portion (2D)	0.65 (0.56–0.72) 0.72 (0.62–0.81)	0.0075 0.0025
LTL dorsal portion (3D) LTL dorsal portion (2D)	0.66 (0.60–0.72) 0.71 (0.61–0.81)	0.0005 0.0005
LTL palmar portion (3D) LTL palmar portion (2D) Trabeculae of the capitate bone Cartilage (triquetrum/hamatum) (3D) Cartilage (triquetrum/hamatum) (2D) Tendon (ECU) (3D) Tendon (ECU) (2D) Median nerve (3D) Median nerve (2D) Ulnar nerve (3D) Ulnar nerve (2D)	0.65 (0.55–0.74) 0.65 (0.59–0.72) 0.87 (0.81–0.91) 0.84 (0.79–0.88) 0.73 (0.65–0.81) 0.73 (0.63–0.83) 0.78 (0.67–0.87) 0.80 (0.72–0.87) 0.82 (0.72–0.91) 0.80 (0.72–0.86) 0.88 (0.78–0.94)	0.022 0.001 < 0.0000001 < 0.0000001 < 0.0000001 0.0005 < 0.0000001 < 0.0000001 < 0.0000001 < 0.0000001 < 0.0000001
Edge sharpness (image quality)	0.74 (0.68–0.80)	< 0.0000001
Perceived tissue contrast (image quality)	0.73 (0.67–0.78)	< 0.0000001
Artefacts (image quality)	0.52 (0.42–0.60)	0.714

*2D*, two-dimensional; *3D*, three-dimensional; *AUC*, area under the curve; *ECU*, extensor carpi ulnaris; *LTL*, lunotriquetral ligament; *SLL*, scapholunate ligament; *TFCC*, triangular fibrocartilage complex; *VGC*, visual grading characteristics

Regarding anatomical visibility, the difference in grading between 7T and 3T was most pronounced for the ulnar nerve (using 2D sequences), where 72% of the cases were graded as a 4 or a 5 (visualization of most anatomical detail or perfect visualization of anatomical detail) for 7T compared to 6% for 3T (Supplementary Table 3). Similarly, at 7T and 3T respectively, grade 4 or 5 was given in 99% versus 56% of cases for trabecular bone, in 79% versus 39% of cases for cartilage (using 2D sequences), in 75% versus 42% of cases for the foveal attachment of the TFCC (using 3D sequences), in 44% versus 19% of cases for the dorsal portion of the LTL (using 3D sequences), and in 69% versus 49% of cases for the dorsal portion of the SLL (using 3D sequences). Regarding image quality, the difference in grading between 7T and 3T was most pronounced for edge sharpness, where 90% of the cases were graded as a 4 or a 5 for 7T compared to 49% for 3T. Regarding anatomical visualization of ligaments, 3D sequences performed better than 2D sequences at both 7T and 3T, receiving a grade of 4 or 5 in a larger proportion of observations. Conversely, 2D sequences were superior to 3D sequences in the visualization of cartilage, nerves, and tendons at both field strengths. For the distribution of all grades given by the four observers, please see Supplementary Table 3.

## Discussion

The current study showed 7T to be superior to 3T in the visualization of anatomical structures of the wrist, as all structures were graded significantly higher for 7T compared to 3T in the VGC analysis. Edge sharpness and perceived tissue contrast were also graded higher at 7T. The proportion of cases with visualization of most anatomical detail, or with perfect visualization of anatomical detail (grades 4 and 5), was higher for all structures at 7T.

Ashman et al in 2002 compared UHF imaging of the wrist (at 8T) with imaging at 1.5T, reporting improved SNR, spatial resolution, and soft tissue contrast [23]. However, while clinically important structures were better visualized, magnetic susceptibility artefacts and chemical shift artefacts were increased. In the current study, there was no significant difference between 7T and 3T regarding artefacts. Commercially available coils for UHF imaging were not available in 2002, and it was suggested that using a phased array coil would increase SNR even further [23]. The technological development of 7T systems in the last decades has allowed wrist imaging at 7T to be significantly improved. It now shows great promise to improve diagnostic confidence and accuracy [10] by delivering excellent delineation of anatomical structures [14]. In contrast to the current work, a previous study revealed no significant difference in the visualization of anatomical structures in the wrist at 7T compared to 3T, despite an increase in SNR of up to 100%, with considerable variation between different anatomical structures [15]. However, Nordmeyer-Massner et al [15] used only one coronal 2D gradient echo sequence, not developed for clinical imaging, and they used a wrist coil array developed for 3T but replicated for operation at 7T, as no commercially available wrist coil for 7T was available at that time. This is in contrast to the current study comparing several sequences developed and optimized for clinical imaging and using commercially available dedicated wrist coils at both field strengths. Interestingly, a cadaver study published in 2011 [16] showed better visibility of articular cartilage surfaces with MR arthrography at 3T compared to 7T. The authors suggested that this difference was due to readers having more experience in evaluating 3T images than 7T images. In addition, sequences were not optimized for 7T because of limited knowledge about tissue and contrast media relaxation parameters [16].

Recently, a study compared MR of the knee at 7T and 3T, in 40 patients with pain of unknown etiology [24]. MR at 7T improved diagnostic confidence, mostly because of higher spatial resolution [24]. The current study demonstrates that anatomic structures in the wrist are better visualized at 7T compared to 3T, and a supposition is that better anatomical visibility and delineation will translate into better detection and definition of pathology. Future studies should be done in patients with wrist pain, to determine if an improvement in diagnostic confidence can be found at 7T compared to 3T, when pathology in the intricate structures of the wrist, such as ligaments and articular cartilage, is suspected. The wrist is a particularly challenging region to depict with MR, due to the small size of clinically important structures such as intercarpal ligaments, the TFCC and articular cartilage [5]. Utilization of a 3D sequence has been reported to enhance visualization of the SLL [25, 26] and the LTL [26], as it allows for MPR in any arbitrarily chosen plane, making it easier to visualize these small, complex, intercarpal structures that should be assessed in several imaging planes [27]. Although in depth comparison between 3D and 2D imaging is beyond the scope of the current study, the distribution of grades in Supplementary Table 3 points in the same direction, with 3D sequences more often receiving the highest grades (grades 4 and 5) regarding visualization of ligaments compared to the 2D sequences for both 7T and 3T.

The small size of the wrist makes it an auspicious area for UHF MR imaging, as it limits RF interference effects such as central brightening [15], which is a key problem in UHF MR brain and abdominal imaging [28, 29]. Tissue heating is generally of greater concern at higher field strengths due to higher transmitted RF energy [30]. This is less of a constraint in wrist imaging, as the extremities are less susceptible to RF power deposition than the head and trunk [30].

Evaluation of how different anatomical structures are visualized on a grading scale is highly subjective, as it relies on human senses and individual interpretation of data. Preference bias and recognition bias are two examples of why the human brain cannot be expected to be able to evaluate images truly objectively. Also, some observers will generally give higher grades than others, which may result in poor inter-observer agreement, even when all observers agree that one test condition is, for example, two steps superior to another. In order to increase conformity in grading, a training session was held with all the observers. To further alleviate these issues, VGC analysis was chosen as the statistical method for this study, as it provides a clear comparison between the two test conditions, without letting subjective interpretation of the grading scale or individual tendencies toward either side of the scale affect the results. Furthermore, it incorporates inter- and intra-observer variations in the results.

Limitations of the current study were the small number of subjects and the lack of pathology in the study population. Also, the 3D sequences were optimized for ligament visualization, which may have resulted in a less optimal visualization of other structures. This is particularly noticeable for tendons and nerves at 3T. However, 7T was superior to 3T in the assessment of these structures using the 2D sequences as well. Additionally, there were slight motion artefacts, affecting edge sharpness, present in several of the data sets at 7T and 3T. Taking into consideration that there was no significant difference in the presence of artefacts between 7T and 3T, these motion artefacts should not have affected the results.

In conclusion, the results show that 7T can improve visualization of anatomical structures of the wrist compared to 3T in healthy volunteers. Further studies are needed to assess if this superiority of 7T MR is evident also in patients with wrist injuries.

## Supplementary Information

ESM 1 (DOCX 31 kb)

## Abbreviations

2D: Two-dimensional
3D: Three-dimensional
AUC_VGC_: Area under the VGC curve
ECU: Extensor carpi ulnaris
FS: Fat saturated
LTL: Lunotriquetral ligament
MPR: Multiplanar reformations
PD: Proton density
RF: Radio frequency
SLL: Scapholunate ligament
SNR: Signal-to-noise ratio
SPACE: “Sampling perfection with application-optimized contrasts using different flip angle evolution”
T: Tesla
TFCC: Triangular fibrocartilage complex
TSE: Turbo spin echo
UHF: Ultra-high field
VGC: Visual grading characteristics

## Declarations

### Guarantor

The scientific guarantor of this publication is Mats Geijer.

### Statistics and biometry

One of the authors has significant statistical expertise.

### Methodology

• diagnostic or prognostic study
