# Supplementary material for: Visualization of wrist anatomy—a comparison between 7T and 3T MRI
Source: Eur Radiol. 2021 Aug 11;32(2):1362–70. doi: 10.1007/s00330-021-08165-5 (PMC8795032; doi:10.1007/s00330-021-08165-5)
Supplement: Supplementary file 1 — (DOCX 31 kb) [file 330_2021_8165_MOESM1_ESM.docx]

Supplementary Table 1. Parameters for the 7T wrist MR protocol

|  | 2D protocol | | | | |  | 3D |
| --- | --- | --- | --- | --- | --- | --- | --- |
|  | PD TSE | | |  | T1 TSE |  | PD TSE |
| **Plane of acquisition** | axial | coronal | sagittal |  | coronal |  | coronal |
| **Repetition time** (ms)  **Matrix size** | 1816  200x225 | 2963  200x225 | 2544  200x166 |  | 300  300x300 |  | 1100  184x181 |
| **Echo time** (ms) | 28 | 28 | 28 |  | 8.8 |  | 110 |
| **Turbo factor** (echo train length) | 7 | 7 | 7 |  | 3 |  | 22 |
| **Receiver bandwidth** (Hz/pixel) | 324 | 324 | 306 |  | 503 |  | 534 |
| **Field of view** (mm^2^) | 70x81 | 70x81 | 70x50 |  | 89x89 |  | 82x82 |
| **Reconstructed voxel dimensions** (mm^3^)  **Aquired voxel dimensions** (mm^3^) | 0.24x0.24x1.2  0.35x0.36x1.2 | 0.24x0.24x1.2  0.35x0.36x1.2 | 0.22x0.22x1.2  0.35x0.36x1.2 |  | 0.28x0.28x2.0  0.30x0.30x2.0 |  | 0.24x0.24x0.24  0.45x0.45x0.50 |
| **Number of slices**  **Phase oversampling**  **Slice oversampling** | 30  No  N/A | 30  No  N/A | 42  No  N/A |  | 15  No  N/A |  | 240  No  10% |
| **In-plane phase encoding direction** | RL | RL | AP |  | RL |  | RL |
| **Fat saturation**  **Partial Fourier faith sampling**  **Acceleration technique** | Yes  No  SENSE 1.4 | Yes  No  SENSE 1.4 | Yes  No  SENSE 1.4 |  | No  7/10  None |  | Yes  No  SENSE 2.2 |
| **Acquisition time** (min:sec)  **Driven Equilibrium** | 3:02  No | 4:50  No | 3:08  No |  | 2:11  No |  | 7:07  Yes |

AP, anteroposterior; MR, magnetic resonance; N/A, not applicable; PD, proton density; RL, right-left; SPACE, “Sampling Perfection with Application optimized Contrasts using different flip angle Evolution”; TSE, turbo spin-echo

Supplementary Table 2. Parameters for the 3T wrist MR protocol

|  | Clinical 2D protocol | | | | |  | 3D |
| --- | --- | --- | --- | --- | --- | --- | --- |
|  | PD TSE | | |  | T1 TSE |  | PD SPACE (GRE) |
| **Plane of acquisition** | axial | coronal | sagittal |  | coronal |  | coronal |
| **Repetition time** (ms)  **Matrix size** (frequency encoding steps x phase encoding steps) | 5580  320x240 | 4070  320x240 | 5050  320x240 |  | 846  320x256 |  | 1100  448x306 |
| **Echo time** (ms) | 26 | 24 | 26 |  | 25 |  | 39 |
| **Turbo factor** (echo train length) | 13 | 13 | 13 |  | 3 |  | 29 |
| **Receiver bandwidth** (Hz/pixel) | 223 | 223 | 223 |  | 233 |  | 385 |
| **Field of view** (mm^2^) | 80x80 | 90x90 | 80x80 |  | 100x100 |  | 220x150 |
| **Reconstructed voxel dimensions** (mm^3^) | 0.3x0.3x2 | 0.3x0.3x2 | 0.3x0.3x3 |  | 0.3x0.3x2 |  | 0.5x0.5x0.5 |
| **Number of slices**  **Phase oversampling**  **Slice oversampling** | 30  40%  N/A | 22  40%  N/A | 25  40%  N/A |  | 15  0%  N/A |  | 112  25%  29% |
| **In-plane phase encoding direction** | AP | RL | AP |  | RL |  | RL |
| **Fat saturation**  **Interpolation**  **Partial Fourier faith sampling**  **Acceleration technique** | Yes  No  No  GRAPPA 2 | Yes  No  No  None | Yes  No  No  GRAPPA 2 |  | No  No  No  None |  | Yes  No  7/8  GRAPPA 2 |
| **Acquisition time** (min:sec) | 3:45 | 5:27 | 2:23 |  | 4:53 |  | 7:05 |

AP, anteroposterior; GRE, gradient-echo; MR, magnetic resonance; N/A, not applicable; PD, proton density; RL, right-left; SPACE, “Sampling Perfection with Application optimized Contrasts using different flip angle Evolution”; TSE, turbo spin-echo

Supplementary table 3. Distribution of scores for anatomical visualization and image quality evaluation given by the four observers in 18 cases, resulting in 72 observations per image evaluation parameter and image set (7T and 3T).

| **Image evaluation parameter** |  | **Distribution of scores given by the four observers in 18 cases (n = 72 observations)** | | | | | | | | | | | | |  |
| --- | --- | --- | --- | --- | --- | --- | --- | --- | --- | --- | --- | --- | --- | --- | --- |
|  | **7T** | | | | | |  |  | **3T** | | | | | |  |
|  | ***Grade*** | | | | | |  |  | ***Grade*** | | | | | |  |
|  | ***1*** | ***2*** | ***3*** | ***4*** | ***5*** | **median** | **%**  **of score**  **4-5** |  | ***1*** | ***2*** | ***3*** | ***4*** | ***5*** | **median** | **%**  **of score**  **4-5** |
| **Anatomical visibility** |  |  |  |  |  |  |  |  |  |  |  |  |  |  |  |
| TFCC radial attachment (3D) | 0 | 2 | 18 | 23 | 29 | 4 | 72% |  | 0 | 4 | 28 | 31 | 9 | 4 | 56% |
| TFCC radial attachment (2D) | 0 | 6 | 19 | 31 | 16 | 4 | 65% |  | 0 | 4 | 39 | 18 | 11 | 3 | 40% |
| TFCC ulnar attachment (3D) | 0 | 2 | 21 | 22 | 27 | 4 | 68% |  | 0 | 8 | 32 | 27 | 5 | 3 | 44% |
| TFCC ulnar attachment (2D) | 0 | 11 | 20 | 26 | 15 | 4 | 57% |  | 0 | 17 | 35 | 15 | 5 | 3 | 28% |
| TFCC foveal attachment (3D) | 0 | 2 | 16 | 29 | 25 | 4 | 75% |  | 0 | 6 | 36 | 26 | 4 | 3 | 42% |
| TFCC foveal attachment (2D) | 1 | 9 | 22 | 30 | 10 | 4 | 56% |  | 0 | 14 | 40 | 15 | 3 | 3 | 25% |
| SLL dorsal portion (3D) | 1 | 1 | 20 | 18 | 32 | 4 | 69% |  | 0 | 9 | 28 | 28 | 7 | 3 | 49% |
| SLL dorsal portion (2D) | 1 | 7 | 18 | 24 | 22 | 4 | 64% |  | 3 | 19 | 25 | 24 | 1 | 3 | 35% |
| SLL palmar portion (3D) | 2 | 11 | 29 | 11 | 19 | 3 | 42% |  | 2 | 17 | 29 | 21 | 3 | 3 | 33% |
| SLL palmar portion (2D) | 9 | 18 | 16 | 19 | 10 | 3 | 40% |  | 19 | 17 | 29 | 6 | 1 | 2.5 | 10% |
| LTL dorsal portion (3D) | 2 | 9 | 29 | 24 | 8 | 3 | 44% |  | 0 | 24 | 32 | 13 | 3 | 3 | 19% |
| LTL dorsal portion (2D) | 12 | 18 | 17 | 23 | 2 | 3 | 35% |  | 23 | 22 | 22 | 3 | 2 | 2 | 7% |
| LTL palmar portion (3D) | 1 | 10 | 25 | 17 | 19 | 3.5 | 50% |  | 3 | 13 | 28 | 23 | 5 | 3 | 39% |
| LTL palmar portion (2D) | 8 | 21 | 17 | 18 | 8 | 3 | 36% |  | 18 | 16 | 23 | 14 | 1 | 3 | 21% |
| Trabeculae of the capitate bone | 0 | 0 | 1 | 24 | 47 | 5 | 99% |  | 1 | 7 | 24 | 32 | 8 | 4 | 56% |
| Cartilage (3D) | 0 | 1 | 18 | 38 | 15 | 4 | 74% |  | 0 | 22 | 37 | 13 | 0 | 3 | 18% |
| Cartilage (2D) | 1 | 4 | 10 | 40 | 17 | 4 | 79% |  | 0 | 10 | 34 | 24 | 4 | 3 | 39% |
| Tendon (3D) | 1 | 13 | 29 | 26 | 3 | 3 | 40% |  | 3 | 30 | 31 | 8 | 0 | 3 | 11% |
| Tendon (2D) | 0 | 4 | 5 | 28 | 35 | 4 | 88% |  | 1 | 3 | 33 | 29 | 6 | 3 | 49% |
| Median nerve (3D) | 3 | 21 | 29 | 11 | 8 | 3 | 26% |  | 15 | 30 | 26 | 1 | 0 | 2 | 1% |
| Median nerve (2D) | 0 | 4 | 20 | 28 | 20 | 4 | 67% |  | 3 | 26 | 29 | 14 | 0 | 3 | 19% |
| Ulnar nerve (3D) | 2 | 23 | 28 | 17 | 2 | 3 | 26% |  | 12 | 38 | 21 | 1 | 0 | 2 | 1% |
| Ulnar nerve (2D) | 0 | 4 | 16 | 36 | 16 | 4 | 72% |  | 1 | 29 | 38 | 4 | 0 | 3 | 6% |
| **Image Quality** |  |  |  |  |  |  |  |  |  |  |  |  |  |  |  |
| Edge sharpness | 0 | 2 | 5 | 46 | 19 | 4 | 90% |  | 0 | 3 | 34 | 31 | 4 | 3 | 49% |
| Perceived tissue contrast | 0 | 0 | 7 | 36 | 29 | 4 | 90% |  | 0 | 2 | 21 | 44 | 5 | 4 | 68% |
| Artefacts | 0 | 3 | 13 | 33 | 23 | 4 | 78% |  | 0 | 2 | 17 | 36 | 17 | 4 | 74% |

2D, two-dimensional; 3D, three-dimensional; LTL, lunotriquetral ligament; SLL, scapholunate ligament; TFCC, triangular fibrocartilage complex
